# Percutaneous Endobiliary Cryobiopsy in Biliary Obstruction: Randomised Study (BICRYOB)

**DOI:** 10.1007/s00270-025-04282-6

**Published:** 2025-12-04

**Authors:** Tomáš Rohan, Barbora Hanžlová, Peter Matkulčík, Jakub Vlažný, Dávid Said, Marek Dostál, Tomáš Andrašina

**Affiliations:** 1 Department of Radiology and Nuclear Medicine, University Hospital Brno and Masaryk University Brno, Jihlavská 340/20, 625 00 Brno, Czechia; 2 Department of Pathology, University Hospital Brno and Masaryk University Brno, Jihlavská 340/20, 625 00 Brno, Czechia; 3 Department of Biophysics, Masaryk University Brno, Kamenice 5, 625 00 Brno, Czechia

**Keywords:** Cryobiopsy, Biliary obstruction, Randomised study

## Abstract

**Design and Purpose:**

To assess feasibility and technical outcome of endobiliary cryobiopsy compared to standard technique of endobiliary forceps biopsy in randomized study.

**Material and Methods:**

This prospective study included 22 patients with indeterminate biliary stenosis. All patients underwent percutaneous endoluminal forceps biopsy and endoluminal cryobiopsy under the fluoroscopy guidance. The order of sample collection was randomized. The technical feasibility of cryobiopsy and the rate of serious complications were analyzed. Sensitivity in detecting malignancy, sample weight, total sample area, sample area without artifacts, and sample quality (five-point Likert scale) assessed by two certified pathologists were compared between both methods.

**Results:**

No CTCAE (Common Terminology Criteria for Adverse Events) v.5 grade 3–4 complications were reported during or after the procedure, and cryobiopsy was technically feasible in all patients. Three of 22 patients were excluded from the analysis. A total of 232 samples were collected (112 forceps biopsy, 120 cryobiopsy). Cryobiopsy and forceps biopsy respectively detected carcinoma in 15/19 patients and 11/19 patients in total yielding positive diagnostic histology for malignancy in 79% and 58% (*p* = 0.11). Among the 17 patients ultimately diagnosed with malignancy, sensitivity for cryobiopsy and forceps biopsy was 88% and 65%, respectively and overall accuracy was 89% (17/19) or cryobiopsy and 68% (13/19) for forceps biopsy. Cryobiopsy provided significantly larger total and artifact-free sample areas (median 2.66 vs 0.84 mm^2^ and 1.77 vs 0.18 mm^2^, respectively; *p* < 0.001), fewer non-evaluable samples (8% vs 40%; *p* < 0,001), and a significantly greater median weight (7.6 vs 3.6 mg; *p* < 0.001). Cryobiopsy samples demonstrated markedly superior quality assessments (median Likert scale value 4 vs 2, *p* < 0.001; Likert > 2 in 83% vs 38%, *p* < 0.001).

**Conclusion:**

Cryobiopsy in the biliary tract appears to be a safe and feasible technique, allowing more representative histological samples to be obtained compared with forceps biopsy.

Level of Evidence: Level 1, Randomized trials.

## Introduction

Biliary tract obstruction is a serious condition that may lead to complications such as hepatic dysfunction, renal failure, nutritional deficiencies, bleeding problems, and infections.

In the case of malignant obstruction, positive histology is crucial in subsequent treatment. Moreover, according to European society for medical oncology, tissue sampling should be obtained before any nonsurgical treatment modality is performed [1]. This could be performed during endoscopic retrograde cholangiopancreatography (ERCP), percutaneous transhepatic cholangiography, or by EUS-guided fine needle biopsy.

Many studies so far compared efficacy, safety and other factors of biliary system sample collection through all possible accesses to increase sensitivity and specificity to maximum [2, 3]. However, obtaining positive histology may be in some cases challenging, and re-biopsies may be needed due to limitations of each technique.

Percutaneous transhepatic biliary drainage and consequent sampling offers an alternative to ERCP, especially in cases where ERCP is challenging or not feasible. While ERCP is often the initial choice, some authors advocate for PTBD as the primary approach in certain situations, such as central biliary tumors [4].

Percutaneous transhepatic biliary access enables either forceps sample collection or brush cytology. Brush cytology usually reaches sensitivity up to 60% and therefore biopsy should be preferred [4]. Reported sensitivity of percutaneous transluminal forceps biopsy (PTFB) in studies with more than 50 patients was 61–78% [5–9], in cross and push technique up to 93% [10, 11]. Metanalysis showed higher diagnostic accuracy of PTFB for intrabiliary malignant origin comparing to extrabiliary malignant origin and for cholangiocarcinoma compared to other tumors [12].

Cryobiopsy offers a promising approach to enhance sample collection sensitivity in biliary tumors. This technique utilizes the cryoadhesive effect to collect appropriate tissue samples. Using CO_2_, the probe temperature decreases to approximately −45 °C (Joule–Thomson effect). This method has been routinely employed in lung biopsies for diagnosing lung tumors and interstitial lung diseases. In these applications, cryobiopsy has demonstrated increased diagnostic rates and larger sample sizes [13–15].

Wirsing et al. conducted the first ex vivo cryobiopsy analysis on explanted tissue from patients undergoing pancreatoduodenectomy, demonstrating significantly larger specimens (5.6 ± 4.5 mm^2^ vs 3.3 ± 5.1 mm^2^) and higher mean histological assessment quality scores compared to gastric and cholangioscopic forceps specimens. Notably, the majority of cryobiopsy specimens were deemed representative [16]. Peveling-Oberhag et al. subsequently reported the first in vivo cholangioscopy-guided cryobiopsy of the biliary system, showcasing a markedly larger specimen obtained from a single patient [17]. To date, however, further studies on cryobiopsy in the biliary system remain limited.

The aim of this prospective randomised, controlled study was to evaluate the feasibility and sensitivity of percutaneous endobiliary cryobiopsy compared to percutaneous endobiliary forceps biopsy in biliary obstruction. We hypothesized that cryobiopsy would yield better sample quality and higher diagnostic accuracy than forceps biopsy.

## Material and Methods

### Study Design

Both biopsies were performed in a single procedure of percutaneous transhepatic cholangiography and the order of biopsies was randomised. Block randomization was used with a block size of 4. The goal was to obtain at least 3 cryobiopsies and at least 3 samples of PTFB. All patients signed informed consent, and the study was approved by hospital review board (number 24-080323/EK). The study was registered on clinicaltrials.gov (NCT06047990). Patients with biliary stenosis of unknown origin referred for percutaneous endobiliary biopsy (not suitable for biopsy under CT or endoscopic control) were included in the study. Inclusion and exclusion criteria are listed in Table 1.

**Table 1:** Inclusion and exclusion criteria

Inclusion criteria
Biliary stenosis of unknown origin indicated for percutaneous endobiliary biopsy (unsuitable for biopsy under CT or endoscopic guidance)
Signed informed consent
*Exclusion criteria*
Histologically verified biliary stenosis
Coagulopathy (INR > 1.5; platelets < 50 000/mL)

### Biopsy Procedure

**Fig. 1:**
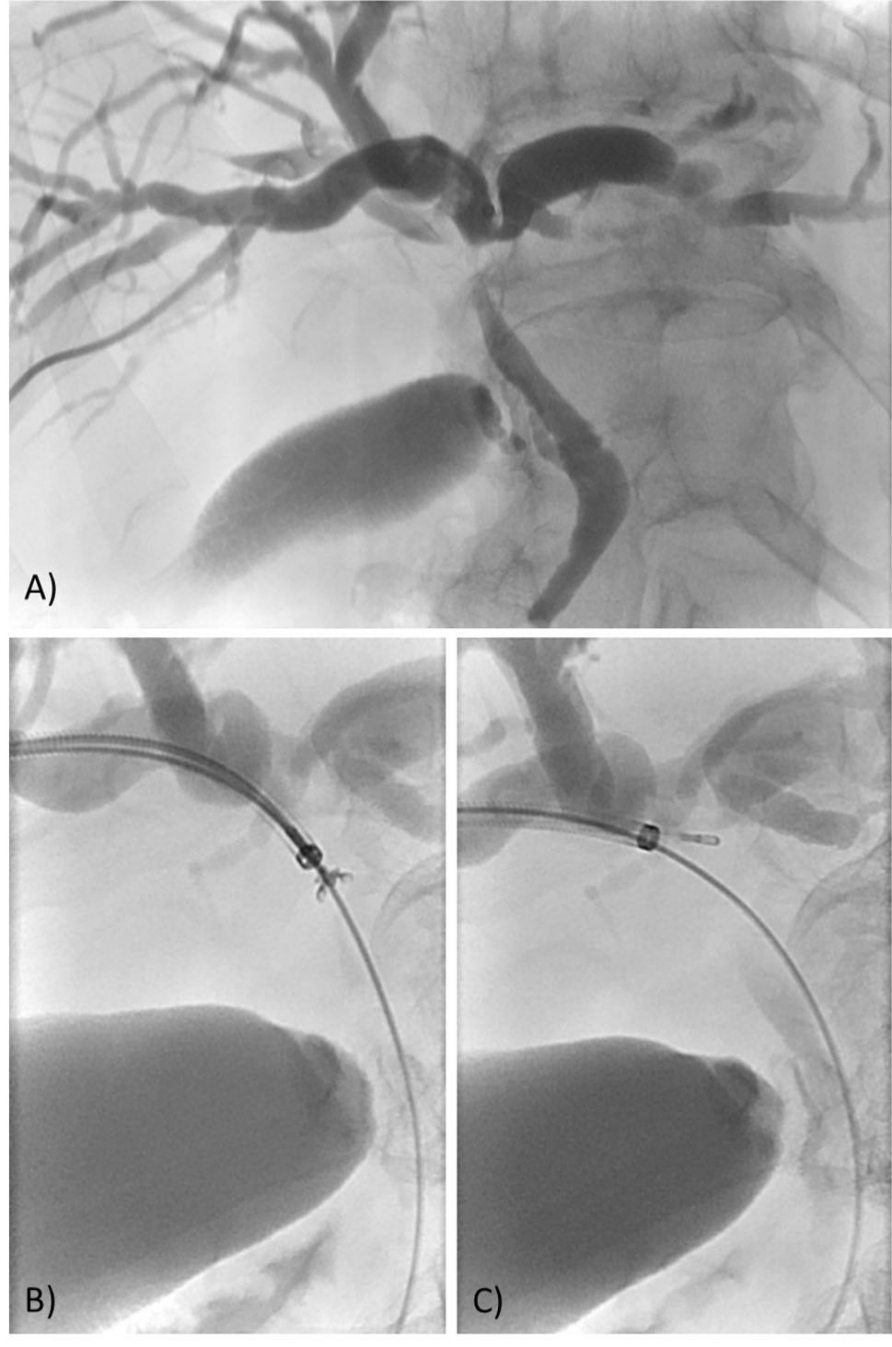
Fluoroscopic image in a patient with Bismuth-Corlette type 1 bile duct stenosis **A**. The stenosis was sampled with 5.2F forceps **B** and a 1.1 mm cryobioptic catheter **C** through a 7F sheath

Biopsy was performed on average 19 days (median 5 days, range 0–221) after the first percutaneous transhepatic biliary drainage (PTBD). After extraction of percutaneous transhepatic drain, over a 0.035" guidewire (Fixed Core Wire Guide, Cook Medical or Starter Guidewire, Boston Scientific) a 9F sheath (Super Sheath R/O, Boston Scientific, USA) was introduced close to the stenosis, and a single-use cryoprobe (Erbe Elektromedizin GmbH, Tübingen, Germany) of a diameter 1.1 mm and length 1150 mm or 7.5F biopsy forceps (MED-111-FOR or MED-190-FOR, both Meditalia, Italy; or Marflow WS-2415BWH, Switzerland) was introduced through the sheath to the stenosis site under fluoroscopic control. In the case of endobiliary biopsy performed during the initial PTBD, a 1.1 mm cryoprobe and 5.2F biopsy forceps were used via a 7F sheath (both Cook Medical, Bloomington, IN, USA) (Fig. 1). The cryoprobe was connected to a cryosurgical device ERBECRYO 2 (Erbe Elektromedizin GmbH, Tübingen, Germany) with a carbon dioxide cylinder, providing sufficient CO_2_ gas pressure for the cryogenic cooling effect. The CO2 gas is directed internally in the cryoprobe to the inside of the distal metal tip. An internal nozzle causes the gas to expand, resulting in a reduction in pressure and hence cooling of the metal tip (Joule–Thomson effect).

We aimed to obtain at least three samples from the stenosis site with each biopsy technique. The maximum number of attempts to obtain a sample was 9. The freezing of the cryoprobe was activated over the footswitch for 4 s before the probe with the sample was removed en-bloc from the patient's body by the operating physician. In accordance with the manufacturer's recommendation, the freezing was kept active by pressing the footswitch until the sample was extracted from the patient's body. Active freezing ensures sufficient cryoadhesion between the cryoprobe and the tissue to prevent loss of the sample in the sheath or biliary tract. Biopsies were performed by the experienced interventional radiologists with 9–25 years of experience (TA, TR) under conscious sedation. The aim was to obtain cryobiopsies and forceps biopsies from the same sites of the stenosis.

Each sample was separately weighed (RADWAG AS 220.R2 plus, Radom, Poland) and placed in a separate vial with formaldehyde. This was followed by histopathological examination.

### Histopathological Evaluation

All tissue samples (cryobiopsies and forceps biopsies) were fixed in formalin, embedded in paraffin and processed for haematoxylin and eosin staining. Histological analysis was performed routinely by a specialised pathologist (JV—7 years of experience). The first objective of the histological analyses was to measure the sample area. Total sample area (tissue without blood contamination) and artifact-free sample area (representative range of total specimen area) were measured in millimetres squared (mm^2^). All samples were scanned using the Zeiss Axio Scan Z1 (Carl Zeiss Microscopy, Germany). The outer borders of the scanned sample were manually outlined, and the area was calculated using Zeiss ZEN 3.1 software (Carl Zeiss Microscopy, Germany).

**Fig. 2:**
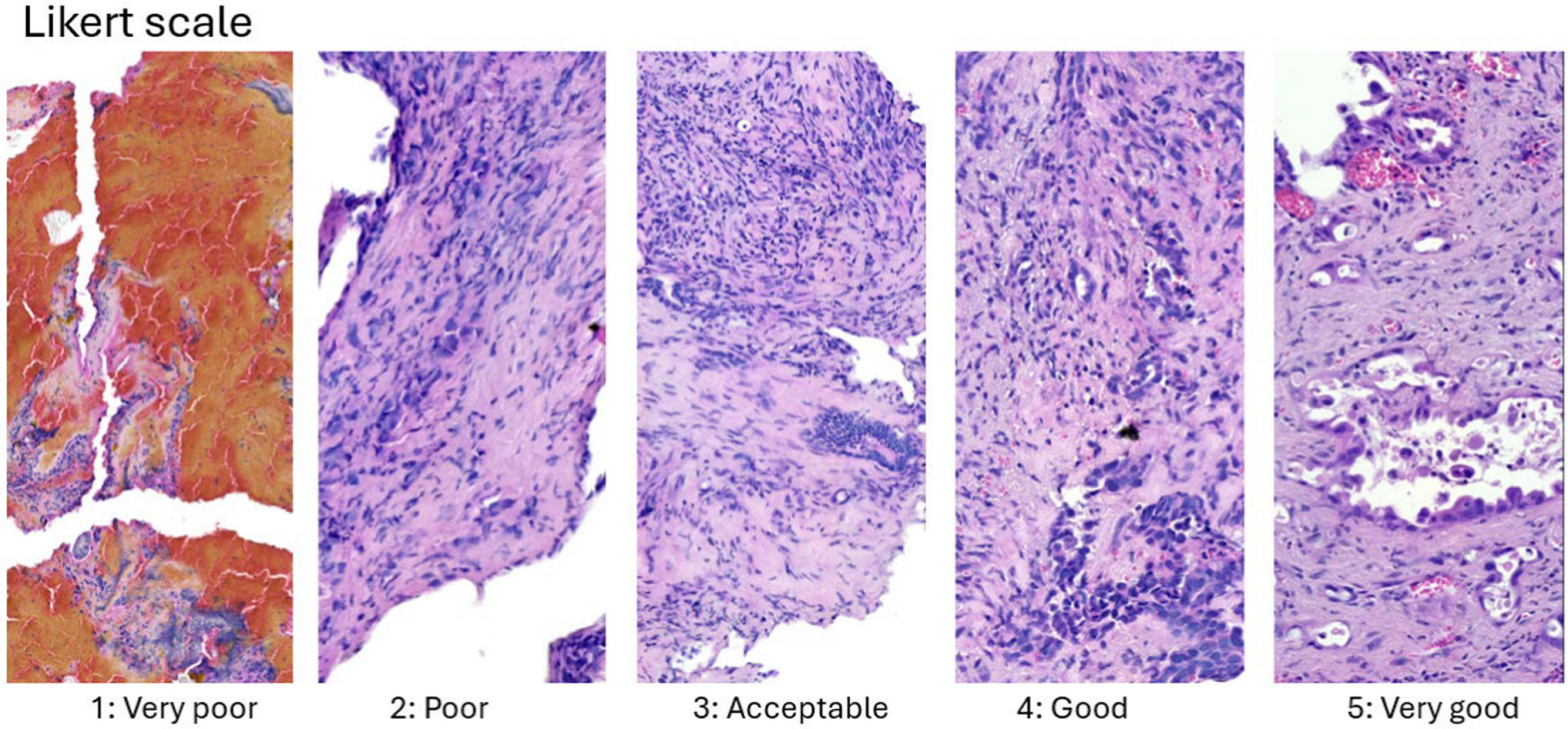
Assessment of sample quality according Likert scale (score 1 = very poor, 2 = poor, 3 = acceptable, 4 = good, 5 = very good)

The second objective of the histological analyses was to assess the quality of the samples. This was performed by two pathologists (JV, DS), both blinded to the biopsy method. A 5-point Likert scale was used to assess sample quality (score 1 = very poor, 2 = poor, 3 = acceptable, 4 = good, 5 = very good) (Fig. 2). The main criterion used to assess the quality of each sample was the presence and quantity of mechanical artifacts.

### Outcome Measures/Study Endpoints

Primary outcome of the study was to assess technical success of cryobiopsy, quality of samples obtained by both methods and a sensitivity of biopsy sampling to detect malignancy. Technical success was defined as the ability to insert a cryoprobe into the stenosis site and collect a sample. Regarding the quality of the samples the weight (in milligrams), total sample area and artifact-free sample area (in mm^2^) were compared between cryobiopsies and forceps biopsies taken during a single procedure. A finding was considered benign if the endobiliary sampled histology was benign and the disease was stable or regressed on imaging methods during 12 months after biopsy. Secondary endpoint included analysis of serious adverse events (grade 3–4 according to CTCAE 5.0).

### Statistical Analysis

For comparison of areas, weights and Likert score between cryobiopsy and conventional forceps biopsy groups respectively Mann–Whitney U-test was used. In case of comparison binary datasets chi-squared test, sensitivity and accuracy were used. For inter-rater reliability assessment based on 5 scaled Likert score quadratic weighted Cohen's Kappa was used. For all statistical calculation IBM SPSS v29 software was used, Bonferroni correction was applied; therefore all statistical tests were on the level of significancy *α* = 0.005.

## Results

**Fig. 3:**
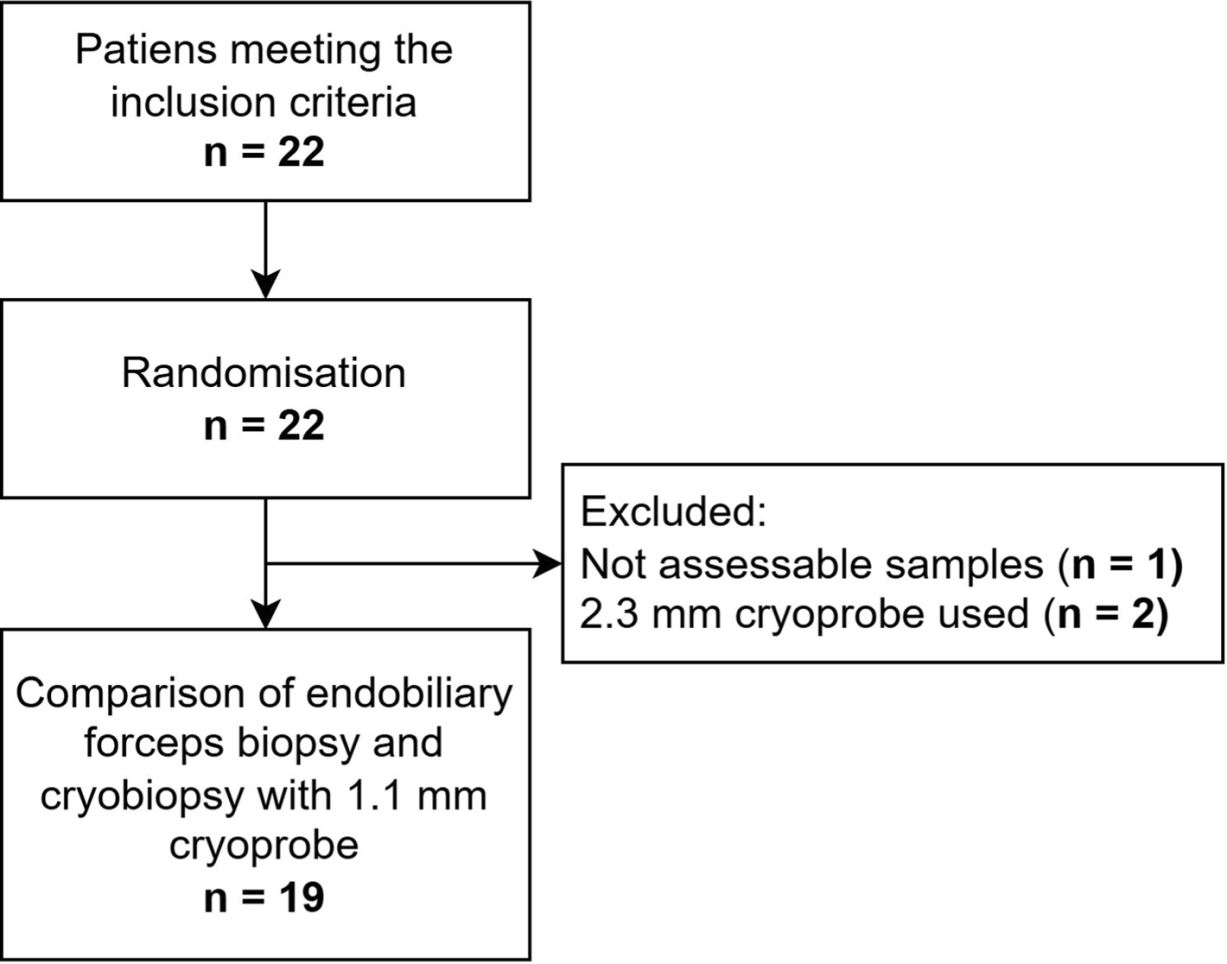
Flowchart demonstrating patients enrolled in the study

All 22 randomized patients met the inclusion criteria. In all patients it was possible to insert the cryoprobe and biopsy forceps into the stenosis site (technical success rate 100%). Three patients were excluded from further analysis: one because none of the obtained samples were histopathologically assessable (due to their insufficient size and unmeasurable dimensions, regardless of collection method), and two because a 2.3 mm cryoprobe was inadvertently used. Therefore, in final evaluation, the study population comprised a total of 19 patients, and the 1.1 mm cryoprobe was utilised in all cases. For forceps biopsy, 7.5F forceps were used in included 16 cases and 5.2F forceps in included 3 cases. No CTCAE v.5 complications of grade 3 or more were observed during the procedures. Flowchart is visualised in Fig. 3. Basic characteristics of patients are listed in Table 2.

**Table 2:** Basic characteristics of included patients

n	19
Female	3 (15.8%)
Age (years)	70.7 ± 8.9
*Localisation and classification of biliary stenosis*	
Bismuth Corlette 1	1 (5.2%)
Bismuth Corlette 2	1 (5.2%)
Bismuth Corlette 3a	2 (10.5%)
Bismuth Corlette 4	8 (42.1%)
Distal common bile duct	5 (21.1%)
Hepaticojejunal anastomosis	1 (5.2%)
Bismuth 2 (benign)	1 (5.2%)
Bismuth 4 (benign)	1 (5.2%)
*Laboratory parameters before biopsy*	Median (min—max)
Billirubin (μmol/L)	100 (8—619)
ALT (µkat/L)	1.18 (0.47—7.85)
AST (µkat/L)	1.13 (0.76—6.44)
GGT (µkat/L)	7.63 (2.29—33.36)
Amylase (µkat/L)	0.46 (0.23—5.93)
Leukocytes (10^9^/L)	10.42 (4.93—15.27)
CRP (mg/L)	45 (3—341)
Hemoglobin (g/L)	120 (92—145)
Thrombocytes (10^9^/L)	317 (88—597)
INR	1.12 (0.86—1.27)

ALT—Alanine transaminase; AST—Aspartate transaminase; GGT—Gamma-glutamyl Transferase; CRP—C-Reactive Protein; INR—International Normalized Ratio

### Sample Characteristics

A total of 232 samples were collected (n = 112 conventional forceps biopsy, n = 120 cryobiopsy). Histopathological concordance between cryobiopsy and forceps biopsy was observed in 15 cases, while discordance occurred in 4 cases. Adenocarcinoma was confirmed in 17 patients and benign biliary stenosis in 2 patients. Cryobiopsy demonstrated adenocarcinoma in 15 patients, while forceps biopsy identified it in 11 (sensitivity 88% and 65%, accuracy 89% and 68% respectively, *p* = 0.11). Cryobiopsy exhibited significantly higher carcinoma detection rates compared to forceps biopsy (60/120 vs 26/112, *p* < 0.001). All carcinomas detected by forceps biopsy were also identified by cryobiopsy. False negative results of cryobiopsy were in case of hilar cholangiocarcinoma Bismuth-Corlette IIIa verified with endobiliary biopsy 14 days later and in case of pancreatic head tumor verified by progression on follow up CT.

**Fig. 4:**
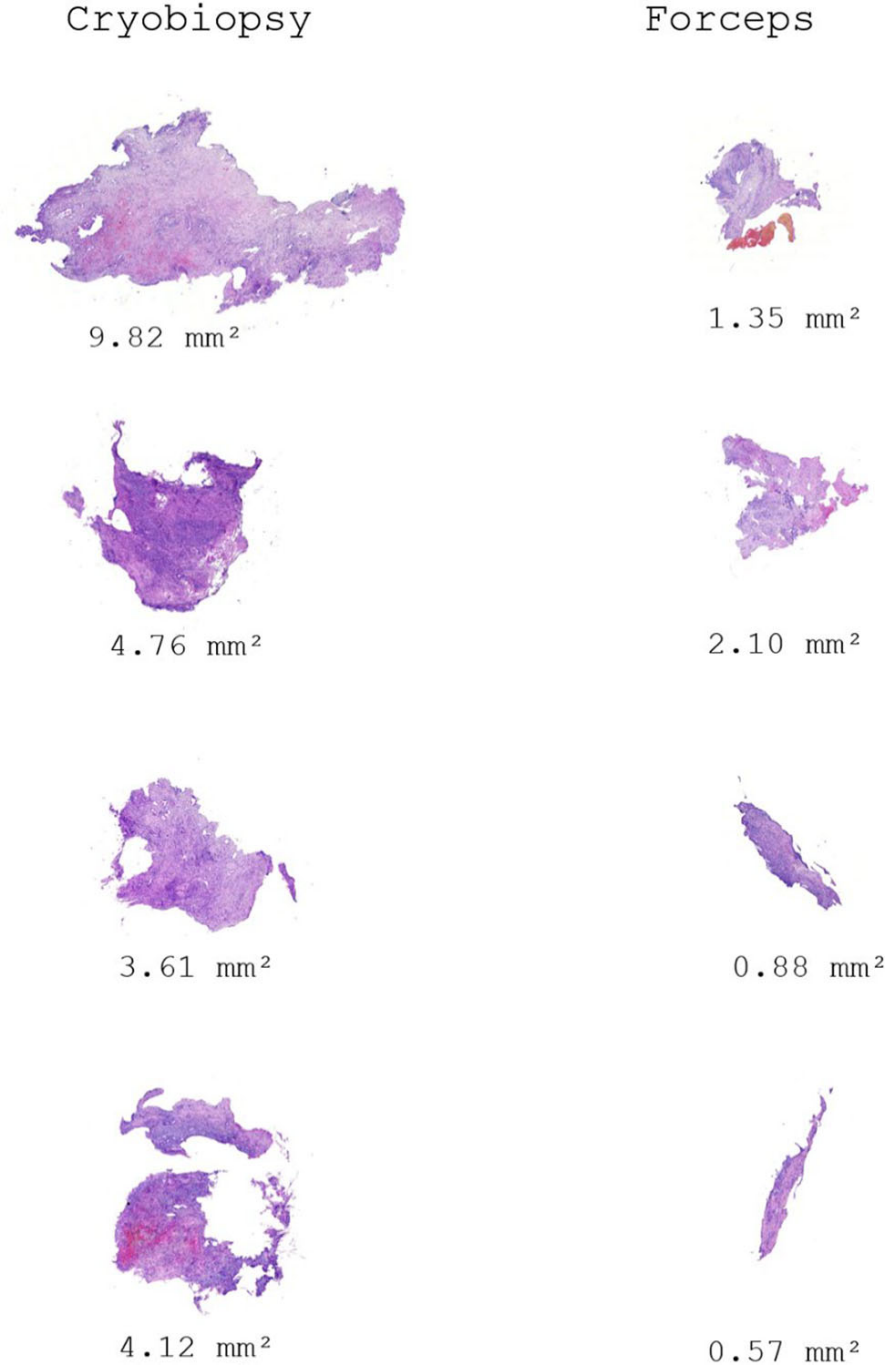
Figure demonstrating differences in total sample area between cryobiopsy using 1.1 mm catheter and 7.5F forceps biopsy of four largest samples in a single patient

Cryobiopsy further provided a significantly larger total sample area (median 2.66 vs 0.84 mm^2^; *p* < 0.001) (Fig. 4), artifact-free sample area (median 1.77 vs 0.18 mm^2^; *p* < 0.001), greater median weight of the samples (7.6 vs 3.6 mg; *p* < 0.001), significantly less number of samples with total sample area lower than 0.01 mm^2^ (1 vs 13%; *p* < 0.001) and samples non-evaluable for artifacts (8 vs 40%; *p* < 0.001).

When subjectively evaluating the quality of the samples according to the Likert scale, cryobiopsy samples achieved significantly better results than the forceps biopsy both in terms of the median. Likert scale value (4 vs 2; *p* < 0.001) and in terms of the frequency of quality samples rated as Likert > 2 (83 vs 38%; *p* < 0.001).

In terms of interrater variability in the assessment of sample quality, there was agreement between the two raters based on quadratic weighted Cohen’s Kappa for forceps biopsy 0.878 and for cryobiopsy 0.862 which represent high reproducibility of both methods.

Detailed results are summarised in Table 3.

**Table 3:** Comparison of cryobiopsy and forceps biopsy results

Final histology			
Benign Malignant	2 17		
Parameter	Cryobiopsy	Forceps biopsy	*p*-value ±
Total number of samples/patients	120/19	112/19	–
Median area [mm^2^] (1st – 3rd quartile)	2.66 (1.32—4.09)	0.84 (0.15—1.50)	< 0.001^+^
Median area without artifacts [mm^2^] (1st – 3rd quartile)	1.77 (0.67—3.19)	0.18 (0—0.81)	< 0.001^+^
Number of samples with area < 0.01 mm^2^	1/120 (1%)	14/112 (13%)	< 0.001 ^‡^
Number of non-evaluable samples (Likert 1)	9/120 (8%)	45/112 (40%)	< 0.001^‡^
Median weight [mg] (1st – 3rd quartile)	7.6 (4 – 12.1)	3.6 (1.4—6.4)	< 0.001
Median Likert score (1st – 3rd quartile)	4 (3 – 5)	2 (1 – 3)	< 0.001
Number of quality samples rated as Likert > 2	100/120 (83%)	42/112 (38%)	< 0.001^‡^
Quadratic weighted Cohen's Kappa (LB—UB)	0.862 (0.814—0.910)	0.878 (0.833 – 0.923)	–
Frequency of samples with malignancy	50.0% (60/120)	23.2% (26/112)	< 0.001^‡^
Sensitivity for malignancy detection [95% CI]	15 of 17 (88%) [0.66 to 0.97]	11 of 17 (65%) [0.41 to 0.83]	0.11^‡^
Overall accuracy for discrimination between benign and malignant disease [95% CI]	17 of 19 (89%) [0.69 to 0.97]	13 of 19 (68%) [0.46 to 0.87]	0.11^‡^

^+^—Mann–Whitney U-test, ^‡^—Chí-squared test, ±—after Bonferroni correction all statistical tests were on the level of significancy *α* = 0.005, LB—Lower Bound, UB—Upper Bound, CI—confidence interval

## Discussion

This study evaluated the feasibility and diagnostic performance of endobiliary cryobiopsy in a randomized, controlled clinical setting. While cryobiopsy is a standard procedure in pulmonology, its application in endobiliary settings has been limited to recent ex vivo and cholangioscopy-guided trials [16, 17]. This technique has several advantages in comparison to conventional forceps sample collection.

Cryobiopsy demonstrated significantly higher detection rate of carcinoma in individual samples (*p* < 0.001), although sensitivity for detection of malignancy in individual patients was not significant (88% and 65% for cryobiopsy and forceps biopsy, (*p* = 0.11). This sensitivity closely approached the maximum reported for forceps biopsy using the cross and push technique (93%) [10]. Between cryobiopsy and forceps biopsy, histopathological findings were consistent in 15 cases and discordant in 4 cases. In all 4 discordant cases, cryobiopsy revealed cholangiocarcinoma while forceps biopsy showed only inflammation or fibrosis. This discrepancy was likely due to the smaller sample size and increased artifacts associated with forceps biopsy. Overall, cryobiopsy detected 33–44% more carcinomas than forceps biopsy (15 vs 11 patients, frequency of samples with malignancy 50.0% vs 23.2%). This can result in earlier diagnosis, more effective treatment and a positive impact on the patient's quality of life.

Our findings regarding the biopsy sample size align with previous ex vivo studies, which demonstrated that cryobiopsy obtains larger tissue samples than endobronchial or gastric biopsy forceps [15, 16]. Additionally, performing radiologist may influence size of specimen by the time of cryobiopsy device activation (based on the manufacturer's recommendation) [18, 19]. In our study, cryobiopsy samples were superior in terms of median area, median weight, Likert scale assessment of quality, and fewer non-evaluable specimens compared to forceps biopsy. Patient may benefit from larger and better-quality samples by enabling subsequent possibility of sequencing (NGS—Next-Generation Sequencing) for targeted therapy [20]. Furthermore, larger sample size and better sample quality could make less demands on the pathologist.

Because of the use of very new instruments other potential disadvantages may appear, specifically unknown compatibility with access sheaths of different sizes. On the other hand, the small size of the 1.1 mm cryoprobe can be a great advantage because it allows to take samples even from very tight or stenosis or through angulated biliary tract, into which conventional 7.5F forceps cannot be inserted or fully opened, and it also allows to perform biopsy under endoscopic control with 3F working channel devices (8-12F endoscope size).

While Peveling-Oberhag et al. utilized cholangioscopy for biopsy guidance, our study employed fluoroscopy alone. However, a study by Onoyama compared the diagnostic sensitivity of peroral cholangioscopy-guided forceps biopsy versus peroral fluoroscopy-guided forceps biopsy for extrahepatic biliary lesions and found no significant difference between the two approaches [21]. It remains unclear whether these findings translate to percutaneous access; however, both a 1.1-mm cryocatheter and smaller 3F forceps can also be used for biopsy under cholangioscopy guidance in the percutaneous setting, which offers the advantage of direct visual control compared with fluoroscopic guidance. Further studies are needed to clarify these aspects.

This study has several limitations apart from above mentioned. The study was based on the analysis of 19 out of 22 recruited patients, yet yielded convincing results regarding sample quality, leading us to stop further enrollment in these settings. Due to the small subset of patients biopsied with 5.2F forceps, all patients biopsied with forceps (5.2F and 7.5F) were analyzed together. Secondly, cryobiopsy was done simultaneously with PTD procedure so potential adverse complication could be attributed either to drainage procedure or cryobiopsy. Due to the routine use of conscious sedation (could mask grade 1–2 complications) and the inherent discomfort of the procedure, only Grade 3 and 4 complications were recorded and analyzed. At the time of procedure patients usually reported less pain during cryobiopsy compared to forceps specimen collection, however again because of ongoing conscious sedation, these results cannot be validly evaluated. Another potential problem may be sample size bias due to their processing, but all patients were processed in the same way and therefore this potential error should not affect the differences between the groups evaluated. Further the sample size may also be affected by the activation time of the cryoprobe and its size. In the study we used the manufacturer's recommended settings in all cases, which are commonly used and well accepted in pulmonology. Longer freezing times could allow larger samples to be obtained but could cause complications. The definitive diagnosis of benign lesions was based on benign histology at endobiliary sampling and follow up for 12 months, which may not be long enough for some slowly progressive malignancies and may be source of interpretative bias.

In the future, endobiliary cryobiopsy should be further analysed in randomised studies across different centers and instruments of various diameters should be tested.

## Conclusion

Cryobiopsy in biliary tract seems to be safe and feasible technique enabling to obtain more representative histology samples in comparison to forceps biopsy. Hence, it shows promising potential to be routinely used in clinical practice.
